# Detection of novel coronavirus (SARS-CoV-2) RNA in peripheral blood specimens

**DOI:** 10.1186/s12967-020-02589-1

**Published:** 2020-11-02

**Authors:** Marjan Azghandi, Mohammad Amin Kerachian

**Affiliations:** 1 Cancer Genetics Research Unit, Reza Radiotherapy and Oncology Center, Mashhad, Iran; 2 Department of Animal Science, Faculty of Agriculture, Ferdowsi University of Mashhad, Mashhad, Iran; 3 Medical Genetics Research Center, Mashhad University of Medical Sciences, Mashhad, Iran; 4 Department of Medical Genetics, Faculty of Medicine, Mashhad University of Medical Sciences, Mashhad, Iran

**Keywords:** COVID-19, SARS, MERS, Serum, Plasma, Diagnosis, Detection

## Abstract

The latest outbreak of pneumonia caused by SARS-CoV-2 presents a significant challenge to global public health and has a major impact on clinical microbiology laboratories. In some situations, such as patients in coma condition, the oropharyngeal or nasopharyngeal sampling is seldom feasible, and blood sampling could be an alternative. In the current article, a comprehensive literature search has been conducted for detecting coronavirus disease 2019 (COVID-19) using plasma or serum samples. To date, twenty-six studies have used SARS-CoV-2 nucleic acid in plasma or serum (RNAaemia) to diagnose COVID-19. The pros and cons are discussed in this article. While the detection of SARS-CoV-2 viral load in respiratory specimens is commonly used to diagnose COVID-19, detecting SARS-CoV-2 RNA in plasma or serum should not lose sight and it could be considered as an alternative diagnostic approach.

## Background

The latest pneumonia epidemic caused by a novel coronavirus (Severe Acute Respiratory Syndrome Related Coronavirus 2; SARS-CoV-2) termed coronavirus disease 2019 (COVID-19) has raised a major risk for global public health. It has had a significant impact on clinical microbiology laboratories over the last few months. Compared to the tests developed for other SARS-CoV diagnosis, the optimal test method to detect COVID-19 is reverse transcription quantitative polymerase chain reaction (RT-qPCR) in respiratory samples [1]. Although respiratory samples have the greatest yield, other samples including stool and blood, could also be used to detect the COVID-19 virus [2, 3], as for other coronaviruses such as SARS and Middle East Respiratory Syndrome (MERS) [4–6]. Numerous studies have used blood samples to diagnose COVID-19 disease, although variable results have been reported (Table 1).

**Table 1.** Studies performed on blood samples for COVID-19 viral RNA detection

Study	Sample types	Detection rate %	RNA extraction method	RNA detection method	Confirmed test	Stage of disease	Refs.
Zheng et al.	Serum	41	MagNA Pure 96	RT-qPCR	Sputum and saliva samples	After admission	[12]
Wölfel et al.	Serum	0	–	RT-qPCR	Oro- or naso-pharyngeal swab specimens	Between 2 and 4 days after the onset of symptoms	[17]
Ling et al.	Serum	0	Magnetic bead-method nucleic acid extraction kit	RT-qPCR	Oropharyngeal swab	After admission	[18]
Zhang et al.	Whole blood and serum	Whole blood: 40 Serum: 20	High Pure Viral RNA Kit (Roche)	RT-qPCR	Oral swabs and anti-SARSr-CoV IgG and IgM ELISA test	After admission	[20]
Wang et al	Blood	1	–	RT-qPCR	Nasopharyngeal swabs	1 to 3 days after hospital admission	[23]
Chen et al	Serum	10.4	–	RT-qPCR	Throat swabs	Immediately after admission	[15]
Chen et al	Blood	10.5	Nucleic Acid Isolation Kit (Da’an Gene Corporation, Cat: DA0630)	RT-qPCR	Anal and pharyngeal swab	_	[16]
Huang et al	Plasma	15	Direct-zol RNA Miniprep kit	RT-qPCR	Laboratory-confirmed 2019-nCoV infection by real-time RT-PCR and next-generation sequencing	_	[13]
Peng et al	Blood	22.2	Nucleic Acid Extraction and Purification Kit (SUPI‐1017; Supbio, Guangzhou, China)	RT-qPCR	Oropharyngeal swabs	After admission	[10]
Lescure et al	Serum	0	Extraction NucleoSpin Dx Virus kit (Macherey Nagel, Düren, Germany)	RT-qPCR	Nasopharyngeal swabs	After hospital admission	[24]
Kujawski et al	Serum	9.09	–	RT-qPCR and whole genome sequencing	Nasopharyngeal swabs and f laboratory-confirmed SARS-CoV-2	After admission	[11]
Chan et al	Serum	16.6	QIAamp Viral RNA Mini Kit (Qiagen, Hilden, Germany)	RT-qPCR	CT scan and laboratory-confirmed SARS-CoV-2	_	[25]

*RT-qPCR* reverse transcriptase quantitative polymerase chain reaction

The review by Wang, distinctly showed that the nucleic acids were presented in the serum or plasma samples for all novel coronaviruses, but the timeout of viremia remained unclear [7]. In another study, Chang et al. [8] suggested that the SARS-CoV-2 RNA was relatively stable in plasma, although it might not indicate any infection.

On February 2, 2020, the Hubei Province Center for Disease Control and Prevention conducted follow-up tests on plasma samples by reporting poor positive results close to the limit of detection. After a week, donor-collected throat swab specimens became rather positive, suggesting the presence of an extremely low viral load in plasma samples [9]. Peng et al. [10] analyzed different patient samples including blood, urine, anal and throat swabs, tested with RT-qPCR. Virus screening in all samples revealed that coronavirus could affect respiratory, digestive, urinary and hematological systems. Thus, clinical symptoms and signs in COVID-19 disease could result from the involvement of different organs. So far, only a few studies have shown that the viral RNA in COVID-19 patients might be detectable in plasma or serum samples (Table 1).

Viral RNA in blood has been detected in COVID-19 patients on the first 2 to 3 days after the onset of symptoms; however, there is no evidence on the plasma and serum viral load during the incubation period [7, 11]. Zheng et al. [12] reported that, from the first week, the load of viral RNA in serum samples gradually increased, followed by a decline in the third week of the disease. In addition, they reported that the rate of viral RNA detection in serum samples was greater in patients with advanced disease than mild cases (45% vs 27%), although the difference was not significant. Since there are limited number of studies indicating the presence of COVID-19 RNA in blood samples, more investigations are needed to determine the time period when the viral RNA is present in blood. Also, it is not clearly known which particular type of patients are linked with the higher occurrence of RNAaemia [13]. According to the experiments carried out to diagnose SARS-CoV-2 RNA in blood samples, on average 10–15% of the critically ill patients have RNA in plasma or serum [7, 10, 14–16]. However, a zero detection rate of viral RNA in serum samples has also been reported [17, 18].

Chen et al. reported that the combination of the substantial level of IL-6 with a meaningful Ct (cycle threshold) value of viral RNA in serum samples can be considered as an effective and accurate biomarker unveiling adverse outcomes [15]. Since the increased IL-6 could be part of a broader cytokine storm that may intensify the outcome, IL-6 could be a possible treatment option for critically ill patients with an uncontrolled inflammatory response [15, 19].

In a recent study, Zhang et al. [20] reported that the current strategy for the detection of viral RNA in oral swabs is not ideal. They indicated that the virus could be present in patients' anal swabs or blood while the oral swab test was negative. Similar studies have also confirmed that the virus can exist in blood or rectal swabs and not to be presented in the throat swab. These patients could act as carriers and spread the disease to other individuals. Thus, it is important to examine samples from different sources to validate the infection [20, 21]. While SARS-CoV-2 RNA has been identified in serum or plasma of infected patients, there is no evidence demonstrating the possibility of SARS-CoV-2 transmission through blood transfusion [22].

## Conclusion

In conclusion, while the detection of SARS-CoV-2 viral load in respiratory specimens is commonly used to diagnose COVID-19, detecting SARS-CoV-2 nucleic acid in plasma or serum should not lose sight. In some situations, such as patients in coma condition, the oropharyngeal or nasopharyngeal sampling is seldom feasible, and thus blood sampling could be an alternative. Moreover, the blood test could have less harm for the hospital staffs who conduct the sampling with the respiratory swaps. It should be noted that the detection of viral RNAaemia might not reveal active infection, but rather at times of post-viral infection. Furthermore, RNA detection by itself does not necessarily indicate the presence of live virus and only patients having positive blood tests with clinical symptoms should receive medical treatment.

However, it remains unclear that whether the RNAaemia is associated with the frequency of cytokine storms or it is related to a particular type of patients. Another challenge for the identification of viral RNA in blood samples may be due to the small amounts of RNA in plasma or serum samples. In addition, large amounts of background RNA can mask the low-abundance of viral RNA fragments leading false negative outcomes. Further investigations in future studies are needed to lower the false negative results. We should keep in mind that although the sampling of oropharyngeal/nasopharyngeal compartments may be challenging, it may not be impossible. This may be justified further as blood specimens may not help in better yields. Once more, further studies are needed.

## Data Availability

Not applicable.
